# Mosquito Mycobiota: An Overview of Non-Entomopathogenic Fungal Interactions

**DOI:** 10.3390/pathogens9070564

**Published:** 2020-07-12

**Authors:** Simon Malassigné, Claire Valiente Moro, Patricia Luis

**Affiliations:** Univ Lyon, Université Claude Bernard Lyon 1, CNRS, INRAE, VetAgro Sup, UMR Ecologie Microbienne, F-69622 Villeurbanne, France; simon.malassigne@etu.univ-lyon1.fr (S.M.); claire.valiente-moro@univ-lyon1.fr (C.V.M.)

**Keywords:** mosquito-microbiota interactions, mycobiota, *Aedes*-*Anopheles*-*Culex* spp

## Abstract

The growing expansion of mosquito vectors leads to the emergence of vector-borne diseases in new geographic areas and causes major public health concerns. In the absence of effective preventive treatments against most pathogens transmitted, vector control remains one of the most suitable strategies to prevent mosquito-borne diseases. Insecticide overuse raises mosquito resistance and deleterious impacts on the environment and non-target species. Growing knowledge of mosquito biology has allowed the development of alternative control methods. Following the concept of holobiont, mosquito-microbiota interactions play an important role in mosquito biology. Associated microbiota is known to influence many aspects of mosquito biology such as development, survival, immunity or even vector competence. Mosquito-associated microbiota is composed of bacteria, fungi, protists, viruses and nematodes. While an increasing number of studies have focused on bacteria, other microbial partners like fungi have been largely neglected despite their huge diversity. A better knowledge of mosquito-mycobiota interactions offers new opportunities to develop innovative mosquito control strategies. Here, we review the recent advances concerning the impact of mosquito-associated fungi, and particularly nonpathogenic fungi, on life-history traits (development, survival, reproduction), vector competence and behavior of mosquitoes by focusing on *Culex*, *Aedes* and *Anopheles* species.

## 1. Introduction

**Figure 1:**
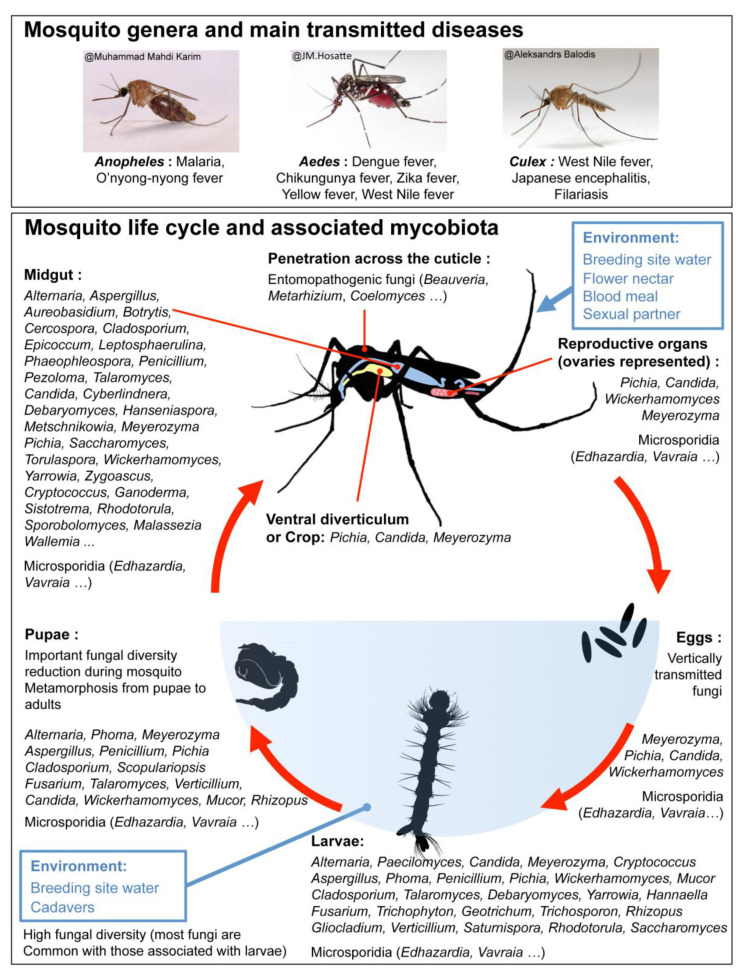
Mosquito-borne diseases with significant public health concern and mycobiota detected along the mosquito life cycle. Given fungal genera are not exhaustive as there are too many genera detected in the adult mosquito (especially in the midgut). To avoid a surcharge of information, only fungal genera detected after gut dissection and/or corresponding to prevalent species (i.e., fungal species found in more than 70% of mosquito individuals) were included. The complete list of fungal genera and species are given in Supplementary Data (see Table S1).

Mosquitoes are insects belonging to the order *Diptera*. They form the family *Culicidae*, which comprise more than 3500 species distributed among 41 different orders [1]. Most species are hematophagous as blood meal is necessary for egg production. Adult females usually feed on vertebrate hosts and use digestive enzymes secreted from their midgut epithelial cells to degrade blood proteins into amino acids required for egg production [2]. Such blood meals can also result in pathogen transmission to humans and other animals. Indeed, when a female mosquito bites an infected host, pathogens are taken together with the blood into the mosquito midgut. After being sucked up in blood, pathogens infect the gut epithelial cells, enter the hemolymph, invade the salivary glands, and the mosquito can then transmit the pathogen while biting a healthy host. [3]. The most important mosquito-borne diseases are transmitted by three mosquito genera (*Anopheles*, *Aedes*, and *Culex*). If *Anopheles* mosquitoes spread malaria parasites (*Plasmodium* spp.) and O’nyong-nyong virus, *Culex* species are major vectors of filarial nematodes and West Nile virus (Figure 1). In addition to nematodes (*Dirofilaria* spp.), *Aedes* mosquitoes transmit several arboviruses, including dengue, chikungunya, West Nile, Zika, and yellow fever viruses [3].

In the past decades, mosquito vectors have expanded their global distribution [4,5], favored by global warming and human activities such as international trade or urban expansion [6,7,8]. This leads to the emergence of mosquito-borne diseases in new geographic areas and causes major public health concerns [3]. In the absence of effective preventive treatments against most pathogens transmitted, vector control remains one of the most suitable strategies against mosquito-borne diseases. The overuse of chemical insecticides in recent years has raised resistance to various molecules in mosquito populations as well as having deleterious impacts on the environment and non-target species [9]. Growing knowledge of mosquito biology has allowed the development of alternative control methods. The mosquito can no longer be considered as an isolated entity and instead should be considered as inseparable from its microbiota with which it interacts and forms a holobiont [10]. This associated microbiota is now recognized to influence many aspects of mosquito biology such as development, physiology, survival, immunity or even vector competence [10,11,12].

Mosquito-associated microbiota is composed of bacteria, fungi, protists, viruses, and nematodes [13,14,15,16,17]. While an increasing number of studies on mosquito-associated microbiota have focused on bacteria, other microbial partners like fungi have been largely neglected [10,12]. Recent studies show the presence of an important fungal diversity in mosquitoes [14,17,18]. Mosquito-associated fungal communities (mycobiota) are mainly composed of Ascomycota (73-92% in *Aedes* and *Culex* species) and Basidiomycota (8–25% in *Aedes* and *Culex* species) including yeasts and filamentous species [14,17,18]. Mosquito-associated Ascomycota species, detected by culture-dependent or -independent methods, belong mostly to the Pezizomycotina and Saccharomycotina subphyla [14,17,18,19,20,21,22,23,24,25,26,27,28]. Among them (see Table S1), several species of filamentous fungi (*Aspergillus gracilis*, *A. puulaauensis*, *Cladosporium* sp., *Phaeophleospora hymenocallidicola*, *Penicillium* sp.) and yeasts (*Aureobasidium pullulans*, *Candida parapsilosis*, *Candida* sp., *Pichia burtonii*) are highly prevalent in mosquito populations and were detected in more than 70% of mosquito individuals [14,16,17,29,30]. Concerning Basidiomycota, mosquito-associated species are mainly affiliated to Agaricomycotina, Ustilaginomycotina and Pucciniomycotina subphyla [14,18,28,29,30,31]. Furthermore, yeasts or yeast-like fungi are an important component as they represent on average 19-47% of the mosquito mycobiota and can even reach 84% in certain *Aedes albopictus* populations [14]. Mosquito larvae acquire their gut mycobiota mainly from the water of breeding sites while adults obtain it from water at emergence as well as from sugar (plants or flower nectars) and/or blood meals for females during their entire life span [29,32,33]. As observed for bacteria, structure and abundance of fungal communities vary according to stage of development with a significant reduction of fungal diversity in newly emerged adults as the midgut undergoes a partial sterilization during metamorphosis from pupae to adult (Figure 1) [32,34]. Blood ingestion by female mosquitoes also induces a reduction of fungal diversity in the midgut by favoring the development of a few species such as *Meyerozyma* spp. [18]. In addition to the midgut, fungi also colonize other mosquito organs such as the ventral diverticulum and reproductive organs [35,36,37].

Mosquitoes and their associated fungi establish different categories of associations ranging along a continuum from parasitic to nonpathogenic interactions. Parasitic interactions include entomopathogenic filamentous fungi, not considered in the present review as they are widely documented because of their potential for mosquito control, and the obligate intracellular parasite microsporidia known to induce negative effects on mosquito biology [24,38,39]. Fungi have also developed commensal or symbiotic relationships with mosquitoes and are then considered as nonpathogenic [32,37]. Knowledge on the role of mycobiota in the development, physiology or immunity of their mosquito host, as well as interference with transmitted pathogens, is henceforth essential to promote the development of new vector control strategies. This review presents key advances and progress in the field of mosquito mycobiota research highlighting the impact of nonpathogenic fungi, including certain microsporidia with non-deleterious effects, on mosquito life-history traits (development, survival, reproduction), vector competence and behavior.

## 2. Influence of Mycobiota on Mosquito Life-History Traits and Digestive Processes

### 2.1. Impact on Development, Survival and Reproduction

As already mentioned, mosquito larvae acquire their gut mycobiota mainly from breeding-sites feeding on fungi that naturally colonize these aqueous environments [32]. It was also demonstrated that female mosquitoes might transmit some bacteria to their offspring through a mechanism of egg-smearing [40]. However, no study has yet investigated whether such a mechanism could also be applied for fungi. Moreover, females may die after laying their eggs and can often be found floating on water surfaces. This provides opportunities for fungi associated with adult mosquitoes to colonize new habitats and larvae that live inside [32]. Microbiota, and by extension mycobiota, is essential for an optimal development of larvae. It was demonstrated that axenic larvae (microbiota-free larvae) exhibit delays in growth of more than six days [41] compared to conventional ones, or do not develop beyond the first instar [42], despite the presence of an excess of sterile food. Similar to other insects feeding exclusively on blood or phloem, the diet of mosquitoes is deficient in several nutrients. In this way, associated fungi like yeasts provide dietary supplementation thanks to their ability to produce essential amino acids, B vitamins, proteins and trace minerals [43]. A recent study has shown that the yeasts *Saccharomyces cerevisiae* and *Pseudozyma* sp. constitute a microbial diet with the highest amounts of proteins and carbohydrates that promotes accumulation of energy reserves (proteins, glycogen, lipids) and development of non-axenic *Aedes aegypti* larvae [44]. Reserve accumulation is essential to allow larvae to reach a critical mass required to complete their metamorphosis to an adult. Concerning *Ae. aegypti,* the minimum critical mass is estimated between 2.7 and 3.2 mg [45]. If larvae that have been fed *S. cerevisiae* or *Pseudozyma* sp. showed a developmental delay of two to three days compared to those fed fish food, 95-100% reached the pupal stage and 85-100% the adult stage [44]. Another study observed variations in terms of survival and development time of *Cx. pipiens* depending on yeast species [32]. Indeed, if *Metschnikowia bicuspidata* and *Wickerhamomyces anomalus* promote survival (70 to 80%) and development of non-axenic *Culex pipiens* larvae (10-15% of larvae achieving their pupal stage), *Cryptococcus gattii* impacts negatively on pupation (no pupae observed) and larval survival (less than 30%) [32].

In addition to their nutritional role, yeasts can also be involved in the induction of gut hypoxia functions in mosquitoes. It was shown that *S. cerevisiae* induces a hypoxia in the gut of *Ae. Aegypti,* working as a signal for growth and molting [42]. By metabolizing carbohydrates into carbon dioxide (CO_2_) and water during aerobic respiration, yeasts reduce gut oxygen levels below 5%. Gut hypoxia activates hypoxia-induced transcription factors (HIFs) that stimulate signal transduction cascade leading to the accumulation of neutral lipids in the fat body and molting [42,46,47]. Such a mechanism could be extended to all mosquito species as the development of axenic *Cx. pipiens* larvae is also promoted by the presence of *S. cerevisiae* [34]. Regarding adult mosquitoes, the microbiota exclusively composed of yeasts maintains a high percentage of survival (68–100%) but reduces their longevity by some days [42,44].

Fungi can also have a significant impact on the fecundity of female mosquitoes, which refers to the number of eggs laid by each female at one time. *Aedes aegypti* females infected by the obligate parasite *Edhazardia aedis* (microsporidia) imbibe 23% less blood and reduce by 30% the number of eggs produced [48]. This negative impact of microsporidia increases with age as *Anopheles gambiae* females infected by *Vavraia culicis* show a reduction in the number of eggs laid of 16% during the first laying and of 45% during the fourth [49]. The localization of the yeast *W. anomalus* in the reproductive organs of both female and male *Anopheles stephensi* mosquitoes may suggest their potential involvement in mosquito reproduction and their probable vertical transmission [37]. The detection of *W. anomalus* in midgut of pre-adult stages (larvae L1-L4 and pupae) and adults that have emerged under laboratory-controlled conditions (i.e., mosquitoes exclusively fed with sterile food and reared in absence of *W. anomalus* in larval breeding water) supports the hypothesis of transstadial transmission. Moreover, the presence of *W. anomalus* in adults up to at least ten days post emergence confirms its ability to persist in the midgut and benefit from nutrients in the mosquito diet [37].

### 2.2. Mutualistic Interactions and Their Role in Digestive Processes

Fungi and mosquitoes mostly establish trophic interactions that improve the acquisition of nutrients and energy for both partners [50]. Both male and female mosquitoes feed on plant nectar, fruit juices, plant sap and honeydew that contain mainly sugars such as glucose, fructose, and sucrose [33]. Following ingestion, a part of these sugars is directly digested by salivary enzymes and assimilated by the mosquito [51]. The other part is stored in the ventral diverticulum (or crop), which is an extension of the foregut near the esophagus and from which several yeasts like *Pichia caribbica* and *Candida etchellsii* have been isolated [35]. Sugars are then progressively transported from the crop to the gut to be digested, absorbed and used as a regular energy source for both the host and active gut microbiota (including fungi) [50,51,52]. In return, the active mycobiota might provide essential nutrients to the mosquito such as amino acids or B vitamins as already observed in other insect models [43]. Differences in the composition of fungal communities assimilating the fructose in *Ae. albopictus* were highlighted according to the sex of mosquito [50]. Whereas *Malassezia* yeasts have been shown to actively metabolize the fructose in males and females, *Cyberlindnera* yeasts as well as the filamentous fungi belonging to *Pezoloma* and *Ganoderma* genera appeared particularly active in females. Moreover, *Aspergillus* and *Cladosporium* genera have been identified as the most active in males [50]. These differences in community composition could be partly explained by competition and synergy phenomena within the fungal community or more generally within the microbiota for fructose assimilation.

Malpighian tubules play key roles in diuresis and detoxification of the uric acid, which is accumulated in these structures within 24 h after a blood meal [53]. In the sand fly *Phlebotomus perniciosus*, the main vector of leishmaniasis in the western Mediterranean area, it was recently showed that the yeast *Meyerozyma guilliermondii* colonizes the midgut of adults and larvae as well as the distal part of the Malpighian tubules of females. Moreover, *M. guilliermondii* possesses an uricolytic activity and presents in its genome the complete uric acid degradation pathway suggesting that this yeast might contribute to the removal of the excess of uric acid after the blood meal of the insect host [54]. Interestingly, *M. guilliermondii* has been also detected in several mosquito species [29,32] and might also be involved in the degradation of the uric acid accumulated in the Malpighian tubules after the blood meal. Other mosquito inhabiting yeasts, such as *W. anomalus*, are able to withstand high concentrations of uric acid and should therefore possess the complete degradation pathway [37].

## 3. Influence of Mosquito-Associated Mycobiota on Vector Competence

### 3.1. Direct Impact through the Production of Fungal Toxins or the Modulation of Enzymatic Activities

Some strains of *W. anomalus* inhabiting the midgut and gonads of *Anopheles* mosquitoes are able to produce lethal toxins that exert a wide spectrum anti-microbial activity and are often referred as to “killer yeasts” [37,55]. This killing mechanism, which could protect *Anopheles* mosquitoes from infection by entomopathogenic fungi, is partly based on an exo-β-1,3-glucanase enzymatic activity of the toxins [56]. Due to their presence in the midgut and the exo-β-1,3-glucanase enzymatic activity of their toxins, these *W. anomalus* strains strongly inhibit the development of *Plasmodium berghei* from gametocytes to ookinetes in females [57]. Parasite death is induced by the hydrolysis of the β-glucans located in cell-wall membranes of parasites. It was shown that yeasts could reduce the number of parasites (zygotes and ookinetes) by 65% [57]. Contrary to the observations made in vitro [56], no parasitic effect has been shown in vivo on oocysts and sporozoites. This lack of effect could be explained by the fact that these two stages of the sporogonic phase are located outside the lumen of the midgut and therefore never come into contact with yeast toxins [57].

Conversely, other fungi can favor pathogen development in mosquitoes. For example, the filamentous fungus *Talaromyces* sp. that naturally colonizes the midgut of *Ae aegypti* promotes mosquito infection with Dengue virus by inhibiting gut digestive enzyme activities and also enhances *Plasmodium* infection in *An. gambiae* [58]. Secretion of heat-sensitive metabolites and/or proteins by the fungus induces the repression of genes encoding several trypsins and endo-proteases involved in the blood meal digestion. In addition, these fungal metabolites were also shown to alter trypsin activity [58].

### 3.2. Indirect Impact through the Modulation of the Immune System

**Figure 2:**
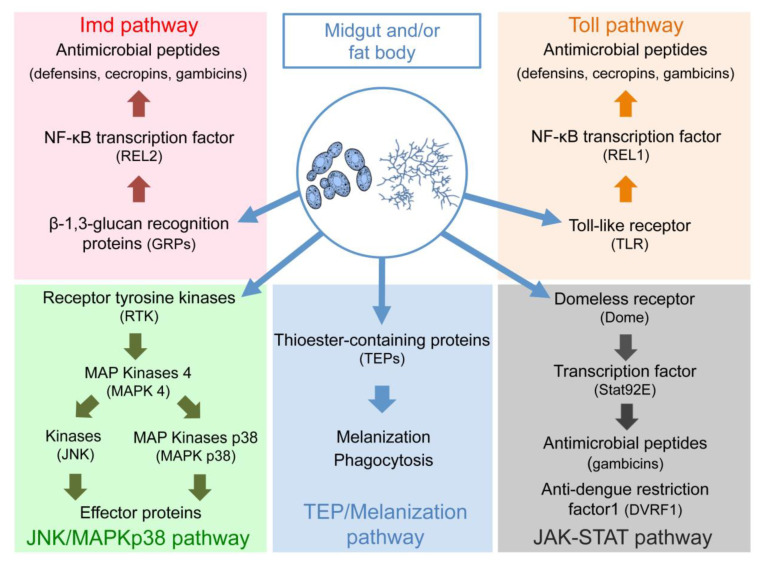
Signaling pathways of mosquito’s innate immunity stimulated by fungi. Fungal surface molecules or secondary metabolites are recognized by specific receptors. This recognition induces the activation of kinases or transcription factors that stimulate the production of antimicrobial peptides or other effector proteins as well as melanization and phagocytosis of fungal cells. Toll, Imd (Immune Deficiency), JAK/STAT (Janus Kinase/Signal Transducer and Activator of Transcription), JNK/MAPKp38 (Jun N-terminal Kinase/Mitogen Activated Protein Kinase p38), TEP (ThioEstercontaining Protein) and immune melanization proteases are the different signaling pathways stimulated by fungi.

Like all insects, mosquitoes only possess innate cellular and humoral immunity, which is a direct response to an infectious agent, nonspecific and not influenced by prior acquired antigen interactions, contrary to adaptive immunity [59]. Innate immunity is based on the recognition of highly conserved molecular patterns restricted to microbes, e.g., MAMPS (Microbe Associated Molecular Patterns), which are recognized by a set of receptors found on the cell surface of host cells, e.g., PRRs (Pattern Recognition Receptors). The ability of fungal partners to stimulate the mosquito immune system has been studied mainly in entomopathogenic fungi [60]. Detection of fungal surface molecules and secreted secondary metabolites by specific receptors in mosquitoes induces the activation of kinases or transcription factors, which stimulate the production of antimicrobial peptides (defensins, cecropins, diptericins, gambicins) or other effector molecules as well as melanization and phagocytosis of fungal cells (Figure 2). Fungi can stimulate different immune signaling pathways in the midgut and/or fat body such as Toll, Imd (Immune Deficiency), JAK/STAT (Janus Kinase/Signal Transducer and Activator of Transcription), JNK/MAPKp38 (Jun N-terminal Kinase/Mitogen Activated Protein Kinase p38), TEP (ThioEster-containing Protein) and immune melanization proteases [60]. The presence of non-entomopathogenic fungi, such as *S. cerevisiae* and *Candida albicans,* in the hemolymph of *Anopheles albimanus* and *Culex quinquefasciatus* mosquitoes induces the melanization of fungal cells after their recognition by proteins containing thioesters (TEPs). This results in the death of fungal cells from lack of nutrients without being phagocytized by hemocytes [61,62]. Fungi may therefore interfere with vector competence by promoting or inhibiting the mosquito immune system. In *An. gambiae*, microsporidia with non-deleterious effects such as *Vavraia culicis* inhibit *P. berghei* development through the stimulation of immune responses such as melanization reaction. It was shown that the number of oocysts in microsporidian-infected mosquitoes is strongly reduced compared to uninfected ones (58% vs. 81%) [63]. Similarly, Microsporidia MB, which is another example of microsporidia with non-deleterious effects, is found in the midgut and ovaries of *Anopheles arabiensis* and alters the development of *Plasmodium falciparum* by stimulating the expression of genes encoding digestive enzymes such as serine proteases, as well as the immune response (cecropins and gambicins) [64]. Strains of *W. anomalus* unable to produce killer toxins reduce the intensity of *P. berghei* infection in *Anopheles* mosquitoes by 38% probably through the activation of the mosquito immune system [57]. Other mechanisms used by fungi to inhibit *Plasmodium* development or viral replication in mosquitoes could involve resource competition and production of cyanide or reactive oxygen species as already observed for bacteria [65,66]. Conversely, the filamentous fungus *Penicillium chrysogenum* makes *An. gambiae* more susceptible to infection with *P. falciparum*. Using arginine for polyamine synthesis, *P. chrysogenum* prevents nitric oxide production, which is considered as the principal anti-*Plasmodium* defense system in the mosquito [67].

## 4. Influence of Fungi and Their Associated Volatile Compounds on Mosquito Behavior

### 4.1. Attractive or Repulsive Effects and Impact on Breeding Site Selection

**Figure 3:**
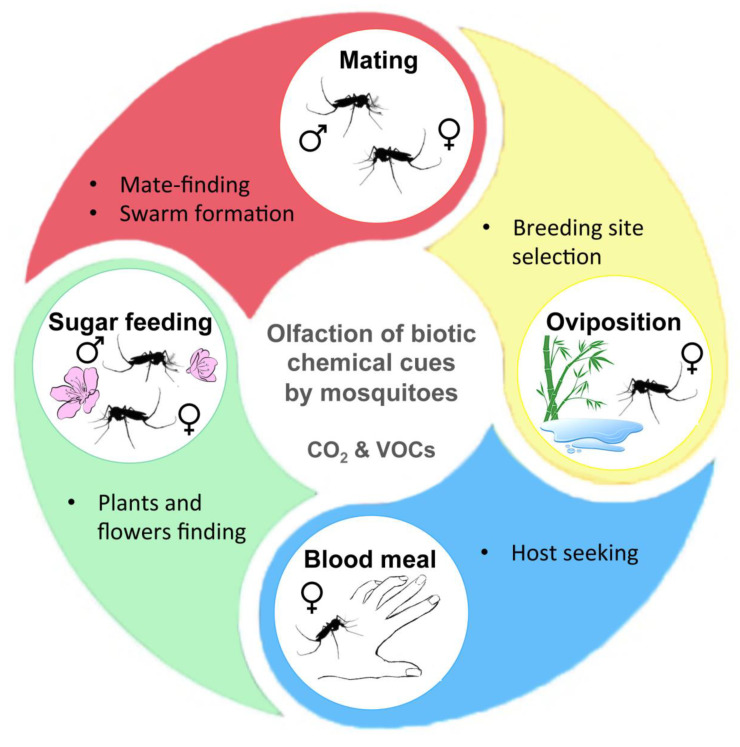
Influence of fungal volatile compounds on mosquito behavior. Mosquitoes use olfactory perception of chemical cues and signals, such as CO_2_ or volatile organic compounds (VOCs), to efficiently find flowering plants, mating partners, vertebrate hosts or breeding sites favorable for larval development. The figure was adapted from Wooding et al. [68] with permission.

Mosquitoes use various signals (visual, humidity, olfactory, etc.) to find their food sources (vertebrate host and/or nectar) and mating partner as well as to locate a place to oviposit (Figure 3) [68]. Chemical cues mainly include CO_2_ and volatile organic compounds (VOCs) able to modulate mosquito behavior such as feeding, mating and egg laying. If plants and vertebrate hosts directly emit some of these chemical volatiles, a part of these compounds is also produced by human or animal skin microbiota, as well as nectar-colonizing microorganisms [69,70]. This is the case of fungal partners, and more particularly yeasts, which produce CO_2_ and volatile secondary metabolites as by-products during fermentation that attract many insects including mosquitoes [33,71,72,73]. In addition to the presence of VOCs, it was demonstrated that CO_2_ produced by yeasts during fermentative metabolism of different carbon sources attracts significantly more mosquitoes than industrial CO_2_ or octenol (fungal aromatic compound) used alone [74,75,76,77]. However, according to the mosquito species, the nature of the VOCs and their concentration, mosquitoes can be attracted as well as repelled [73]. Even if the fermentation of honey by yeast produces higher amounts of VOCs, including many attractive compounds such as hexanoic acid or phenylethyl alcohol, sucrose and molasses are more attractive to mosquitoes. In that case, the absence of some VOCs with repellent properties could favor mosquito attraction [73]. Fungal spores, such as those of the entomopathogenic species *Beauveria bassiana* and *Metarhizium anisopliae,* attract *An. stephensi* females by producing VOCs that have not been yet identified [78].

Gravid mosquito females assess the suitability and accessibility of oviposition sites by physical cues and semio-chemicals released from larvae, eggs and/or of microbial origin [68]. Gravid *Ae. aegypti* females prefer breeding sites that contain eggs of the same species and larvae that are not starving or not infected with a deleterious parasite [79]. *Aedes aegypti* mosquitoes are naturally colonized by the yeast *Candida pseudoglaebosa* and it was shown that the presence of this yeast in the water of breeding sites attracts gravid females for oviposition [79]. Conversely, *S. cerevisiae*, which is not a member of the mosquito gut mycobiota, does not seem to attract ovipositing *Cx. pipiens* females to the breeding site [34]. It was also demonstrated that secondary metabolites produced by the saprotrophic fungus *Trichoderma viride* would attract 76% of gravid *Cx. quinquefasciatus* females [80]. Similarly, the two filamentous fungi *Fusarium fujikuroi* and *Fusarium falciforme* known to colonize rhizomes of the grass *Cyperus rotundus,* which is found in natural *Anopheles* larval habitats, are able to emit VOCs and in particular cedrol that attracts gravid *An. gambiae* females [81,82].

### 4.2. Impact on Larval and Adult Feeding Behavior

As mentioned above, mosquitoes are attracted by CO_2_ and VOC emissions from plant mycobiota and more particularly nectar-inhabiting yeasts [33,70]. These fungal volatile compounds signal the presence of sugar sources to the insect and allow yeasts to be dispersed during insect foraging [71,72]. This behavior explains the presence in the mosquito mycobiota of many phytopathogenic fungi and yeasts known to inhabit floral nectar [14,17]. Microsporidia infection impacts the frequency of blood meal in gravid females. For example, *Edhazardia aedis* strongly reduce the amount of blood ingested by *Ae aegypti*, females as well as the number of laid eggs [48]. As previously stated, yeasts are able to promote larval development through nutrient intake and energetic accumulation in the mosquito *Ae. aegypti* [44]. Interestingly, in *Anopheles* mosquitoes, it was also demonstrated that larvae responded to the presence of yeasts recognized as potential behaviorally active odorants [83,84]. Contrary to the insect repellent DEET, *S. cerevisiae* slow down the average velocity of movements of larvae as well as their body rotations while increasing the rest time [84].

## 5. Conclusions

Mosquito population control is an essential step for arboviral disease transmission management. Compared to the overuse of insecticides, the identification and utilization of associated-fungi that could reduce mosquito development and arbovirus transmission or impact their behavior might be an environmentally friendly strategy for controlling vector-borne diseases. Despite the growing number of studies concerning the impact of nonpathogenic fungi on the biology of mosquitoes, they are still scarce. Moreover, these studies concern only few fungal species including the yeast *S. cerevisiae*, which is not a member of the mosquito gut mycobiota. Additionally, most studies focused on *An. gambiae*, *Cx. pipiens*, *Cx. quinquefasciatus* and *Ae. aegypti* mosquitoes and largely ignored the tiger mosquito *Ae. albopictus* considered nowadays as one of the most invasive species. Further knowledge concerning the impact of nonpathogenic fungi on life-history traits, vector competence and behavior of mosquitoes is henceforth essential to be able to develop new vector control strategies. For example, yeasts are often used in attractive baits to generate biogenic CO_2_. Better knowledge of yeasts and their associated emitted VOCs might improve these techniques and open new avenues for the development of efficient mosquito control methods.

## Supplementary Materials

The following are available online at https://www.mdpi.com/2076-0817/9/7/564/s1, Table S1: Overview of mosquito-associated fungal species found either in laboratory or field populations. Entomopathogenic fungi and obligate intracellular parasites (Microsporidia) are not included. All fungal species have been identified, using culture-dependent or -independent methods, either after mosquito surface sterilization only or after internal organ dissection.
